# *Rubrivirga aquatilis* sp. nov. and *Rubrivirga halophila* sp. nov., isolated from Korean coastal surface seawater

**DOI:** 10.71150/jm.2504017

**Published:** 2025-08-13

**Authors:** Jisoo Han, Yeonjung Lim, Mirae Kim, Jang-Cheon Cho

**Affiliations:** Department of Biological Sciences and Bioengineering, Inha University, Incheon 22212, Republic of Korea

**Keywords:** *Rubrivirga aquatilis*, *Rubrivirga halophila*, polyphasic taxonomy, marine bacteria, novel species, genome

## Abstract

Two Gram-stain-negative, obligately aerobic, non-motile, short rod-shaped bacteria, designated IMCC43871^T^ and IMCC45206^T^, were isolated from coastal surface seawater collected from the Yellow Sea and the South Sea of Korea, respectively. The two strains shared 99.2% 16S rRNA gene sequence similarity with each other and exhibited ≤ 98.4% similarity to three described *Rubrivirga* species. Average nucleotide identity (ANI) and digital DNA–DNA hybridization (dDDH) values between IMCC43871^T^ and IMCC45206^T^ were 88.5% and 36.3%, respectively, confirming that they represent two distinct species. Their ANI (≤ 77.7%) and dDDH (≤ 21.4%) values relative to the type strains of the genus *Rubrivirga* further supported the recognition of strains IMCC43871^T^ and IMCC45206^T^ as two novel species within the genus. The complete genomes of IMCC43871^T^ (4.17 Mb, 71.8% G + C content) and IMCC45206^T^ (4.17 Mb, 72.8% G + C content) fall within the known genomic range of the genus. Cellular fatty acid, quinone, and polar lipid profiles were consistent with the chemotaxonomic features of the genus *Rubrivirga*, supporting their affiliation with the genus. Based on phylogenetic, genomic, and phenotypic evidence, strains IMCC43871^T^ and IMCC45206^T^ are proposed as two novel species, *Rubrivirga aquatilis* sp. nov. and *Rubrivirga halophila* sp. nov., respectively. The type strains are IMCC43871^T^ (= KCTC 102072^T^ = NBRC 116463^T^) and IMCC45206^T^ (= KCTC 92925^T^ = NBRC 116172^T^ = CCTCC AB 2023136^T^).

## Introduction

The genus *Rubrivirga*, a member of the family *Rubricoccaceae* within the class *Rhodothermia*, currently comprises three validly published species: *Rubrivirga marina* (Park et al., 2013), the type species; *Rubrivirga profundi* (Song et al., 2016); and *Rubrivirga litoralis* (Rey-Velasco et al., 2024). *R. marina* SAORIC-28^T^ was isolated from deep seawater (3,000 m) in the western Pacific Ocean, followed by *R. profundi* SAORIC-476^T^ from mesopelagic seawater (500 m) in the same region. The most recently described species, *R. litoralis*, was obtained from microcosms of coastal surface seawater collected in Blanes Bay, located in the northwestern Mediterranean Sea. Members of the genus *Rubrivirga* are Gram-stain-negative, non-motile, aerobic or facultatively anaerobic bacteria that exhibit pale red pigmentation and typically grow under mesophilic and slightly halophilic conditions. The genus is characterized by menaquinone-7 (MK-7) as the major respiratory quinone and phosphatidylethanolamine (PE), phosphatidylglycerol (PG), and diphosphatidylglycerol (DPG) as predominant polar lipids. The genome sizes (3.72–4.98 Mb) and DNA G + C contents (71.5–73.7 mol%) of the three described species show a relatively narrow range, indicating genomic coherence within the genus.

*Rubrivirga* species are known to contribute to marine ecosystems by catabolizing dimethylsulfoniopropionate (DMSP), a key process in sulfur cycling and climate regulation, and by degrading organic matter and detoxifying sulfide in mangrove environments (Rey-Velasco et al., 2024; Wainwright et al., 2023). Notably, *R. marina* encodes a PL6 family alginate lyase (AlyRm1) with broad substrate specificity and high alkaline tolerance, suggesting ecological adaptation to marine environments and potential for biotechnological applications (Zheng et al., 2023). However, despite the ecological relevance of this genus, only one of the three currently described *Rubrivirga* species originates from a coastal environment, and their distribution along coastal gradients remains largely unexplored. The Korean coastline, shaped by dynamic estuarine mixing and pronounced seasonal variability, harbors diverse microbial assemblages structured by sharp gradients in salinity, nutrients, and organic matter (Han et al., 2022). In recent years, numerous novel bacterial species have been continuously isolated and described from Korean coastal environments, reflecting the high microbial diversity harbored by these dynamic ecosystems (Lee et al., 2024; Tak et al., 2024; Yang et al., 2023, 2024).

As part of a broader survey of prokaryotic diversity in island-associated marine habitats, two strains, designated IMCC43871^T^ and IMCC45206^T^, were isolated and characterized from Korean coastal seawater. Comprehensive phenotypic, phylogenetic, chemotaxonomic, and genomic analyses revealed that these strains represent two novel species of the genus *Rubrivirga*. This finding expands the ecological and taxonomic breadth of the genus *Rubrivirga*.

## Materials and Methods

### Isolation and culture condition

Strain IMCC43871^T^ was isolated from coastal surface seawater collected at Daebu Island, Ansan (37°16'58.0"N 126°29'13.0"E) in June 2022, and strain IMCC45206^T^ was obtained from Somaemul Island, Tongyeong (34°37'20.8"N 128°32'55.7"E), Republic of Korea in October 2022. Seawater samples were serially diluted and spread onto marine agar 2216 (MA; Difco). After incubation at 20°C for 5 d, single colonies were streaked and purified through three successive transfers. Following determination of optimal growth temperatures, strains IMCC43871^T^ and IMCC45206^T^ were routinely cultured on MA at 25°C and preserved as 10% (v/v) glycerol suspensions at –80°C. Anaerobic growth was tested using a commercial gas-pack system (AnaeroPack; Mitsubishi Gas Chemical) at 25°C. For comparative phenotypic characterization under identical culture conditions, the closely related type strains *R. marina* KCTC 23867^T^ and *R. profundi* KACC 18401^T^ were obtained from the Korean Collection for Type Cultures (KCTC) and the Korean Agricultural Culture Collection (KACC), respectively, and cultivated on MA at 25°C for 7 d.

### Phylogenetic analysis based on 16S rRNA gene sequences

Genomic DNA from strains IMCC43871^T^ and IMCC45206^T^ was extracted using the DNeasy PowerSoil Kit (Qiagen) according to the manufacturer's instructions. Amplification of the 16S rRNA genes was performed using the universal bacterial primers 27F and 1492R (Weisburg et al., 1991), and sequencing was conducted by Biofact Co. (Korea) using the Sanger method. The resulting sequences, comprising 1,428 bp for IMCC43871^T^ and 1,455 bp for IMCC45206^T^, were analyzed using BLASTn against the GenBank database and pairwise sequence comparisons were performed using the EzBioCloud server (Yoon et al., 2017) to determine their phylogenetic affiliations.

For phylogenetic analyses, the 16S rRNA gene sequences of the two strains and their closest relatives were aligned using the SILVA Incremental Aligner and imported into the ARB software package (Ludwig et al., 2004). Aligned sequences were then exported from ARB and used to construct phylogenetic trees with MEGA X (Kumar et al., 2018) to determine the phylogenetic positions of the strains. Three tree-building algorithms were employed: maximum likelihood (ML) based on the Tamura-Nei model (Felsenstein, 1981), neighbor-joining (NJ) using the Jukes-Cantor model (Saitou and Nei, 1987), and minimum-evolution (ME) also using the Jukes-Cantor model (Fitch, 1971). Tree topologies were evaluated by bootstrap analysis with 1,000 replicates (Felsenstein, 1985).

### Genome sequencing and analysis

Whole-genome sequencing was performed using the Oxford Nanopore Technologies (ONT) platform with a Flongle flow cell (R9.4.1), following the protocol provided with the Ligation Sequencing Kit (SQK-LSK109). In parallel, short-read sequencing was conducted using a 2 × 150 bp paired-end strategy on the Illumina NovaSeq 6000 platform (DNA Link Corp., Korea). Raw ONT signal data were basecalled using Dorado v7.2.13, and adapter sequences were removed with Porechop v0.2.4. The filtered long reads were assembled using Hybracter with the --medakaModel r941_min_sup_g507 option (Bouras et al., 2024). Genome completeness and contamination were assessed using CheckM v1.1.3 (Parks et al., 2015).

For comparative genomic analyses and assessment of genome relatedness, the genome assemblies of *R. marina* SAORIC-28^T^ (accession no. MQWD00000000) and *R. profundi* SAORIC-476^T^ (MVOI00000000) were retrieved from the GenBank database. Average nucleotide identity (ANI) and digital DNA–DNA hybridization (dDDH) values were calculated using JSpeciesWS (Richter et al., 2016) and the Genome-to-Genome Distance Calculator (GGDC 3.0) (Meier-Kolthoff et al., 2013), respectively. To construct a genome-based phylogenetic tree, 81 conserved bacterial core genes were extracted using the UBCG2 pipeline (Kim et al., 2021) and analyzed with RAxML v8.2.12 (Stamatakis, 2014). Functional genome annotation was performed using Prokka (Seemann, 2014), and the predicted protein sequences were assigned to KEGG orthology terms via the BlastKOALA tool (Kanehisa et al., 2016). In addition, the distribution of functional categories based on clusters of orthologous groups (COG) was assessed by querying the protein sequences against the COG database using RPS-BLAST (e-value cutoff: 0.01) (Marchler-Bauer et al., 2013).

### Physiological and chemotaxonomic analysis

Cell morphology was observed using transmission electron microscopy (CM200, Philips) with cells negatively stained using 1% (w/v) uranyl acetate on carbon-coated copper grids (Electron Microscopy Sciences). The Gram reaction was determined using the KOH-based non-staining method (Powers, 1995). Catalase activity was tested by applying a 3% (v/v) hydrogen peroxide solution, and oxidase activity was assessed with 1% (w/v) Kovac’s reagent (bioMérieux). Bacterial motility was evaluated by stabbing into marine soft agar containing 0.5% (w/v) agar. Growth temperature range and optimum were determined on marine agar (MA) at 4°C, 10–30°C (at 5°C intervals), 37°C, and 42°C. Salt tolerance was tested on NaCl-free MA supplemented with NaCl at final concentrations ranging from 0% to 20% (w/v), in 0.5% increments from 0% to 4%, and at additional concentrations of 5%, 7.5%, 10%, 15%, and 20%. The pH range and optimum for growth were assessed in marine broth adjusted to pH 5.0–10.0 (in 1.0-unit intervals), using appropriate buffer systems: citrate (pH 4.0), MES (pH 5.0), MOPS (pH 6.0), HEPES (pH 7.0), and CHES (pH 8.0–11.0). Hydrolytic activities were evaluated on MA supplemented with the following substrates: starch (1%, w/v), colloidal chitin (1%, w/v), casein (3% skim milk, w/v), CM-cellulose (1%, w/v), Tween 20 (1%, v/v), and Tween 80 (1%, v/v). DNA degradation was tested on DNase test agar (BD Diagnostics) supplemented with 2% (w/v) NaCl. Hydrogen sulfide (H₂S) production was assessed using triple sugar iron (TSI) agar (BD Diagnostics) containing 2% (w/v) NaCl. Additional biochemical characteristics were determined using API 20NE and API ZYM strips (bioMérieux), following the manufacturer’s protocols with medium salinity adjusted to 2% (w/v) NaCl.

Fatty acid methyl ester (FAME) analysis was performed using biomass harvested from strains IMCC43871^T^, IMCC45206^T^, *R. marina* SAORIC-28^T^, and *R. profundi* SAORIC-476^T^ grown on MA at 25°C for 7 d. FAME profiles were obtained by gas chromatography (Agilent 7890 GC) and interpreted using the Sherlock Microbial Identification System (MIDI), version 6.1, with the TSBA6 database (Sasser, 1990). Polar lipids were extracted according to the method of Minnikin et al. (1984) and separated by two-dimensional thin-layer chromatography (TLC) on silica gel 60 F_254_ plates. Total polar lipids were visualized using molybdatophosphoric acid, and functional group-specific detection was performed with ninhydrin (aminolipids), molybdenum blue (phospholipids), *α*-naphthol (glycolipids), and Dragendorff’s reagent (phosphatidylcholine). Respiratory quinones were analyzed by reverse-phase partition chromatography using Merck HPTLC RP-18 F_254_ plates following the method of Collins et al. (1980).

### Nucleotide sequence accession numbers

The 16S rRNA gene sequences of strains IMCC43871^T^ and IMCC45206^T^ have been deposited in the GenBank/EMBL/DDBJ database under accession numbers PV186484 and PV076729, respectively. The complete genome sequences of IMCC43871^T^ and IMCC45206^T^ are available under accession numbers CP177355 and CP177357, respectively.

## Results and Discussion

### 16S rRNA gene-based phylogeny

**Fig. 1.**
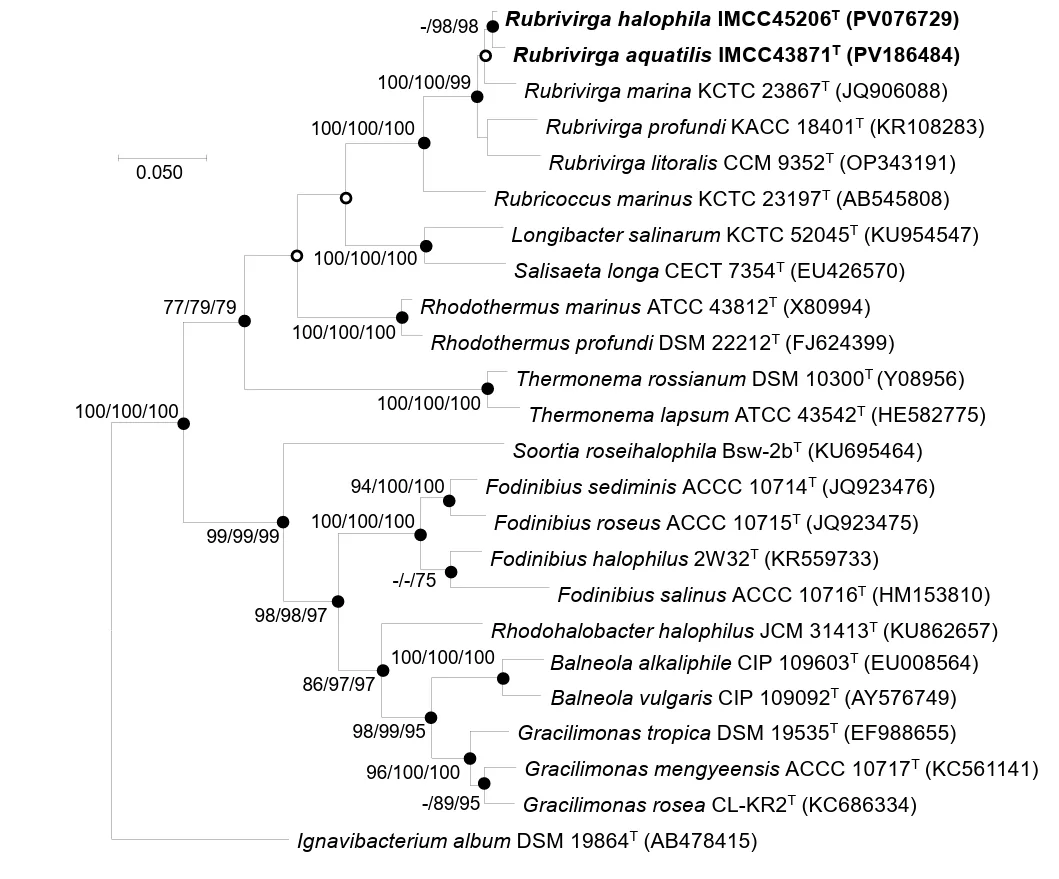
Maximum-likelihood phylogenetic tree based on 16S rRNA gene sequences showing the positions of strains IMCC43871^T^ and IMCC45206^T^. Bootstrap values (expressed as percentages of 1,000 replications) over 75% are shown at nodes for maximum-likelihood, neighbor-joining, and minimum-evolution methods, respectively. Filled circles indicate that the corresponding nodes were recovered by all treeing methods. Open circles indicate that the corresponding nodes were recovered by any two out of three. Bar, 0.05 substitutions per nucleotide position.

The 16S rRNA gene sequence similarity and phylogenetic analyses revealed that strains IMCC43871^T^ and IMCC45206^T^ are affiliated with the genus *Rubrivirga*. The two strains shared 99.2% 16S rRNA gene sequence similarity, which is slightly above the generally accepted species delineation threshold of 98.7% (Chun et al., 2018; Kim et al., 2014). Given this high level of 16S rRNA gene sequence similarity between the two strains, classification of the two strains as separate novel species required further genomic demarcation. Strains IMCC43871^T^ and IMCC45206^T^ showed the highest sequence similarity to *R. marina* SAORIC-28^T^ (98.1% and 98.4%, respectively), followed by *R. profundi* SAORIC-476^T^ (97.8% and 97.9%) and *R. litoralis* F349^T^ (97.1% and 97.0%). Since the 16S rRNA gene sequence similarities between the two strains and the validly published *Rubrivirga* species were all below the 98.7% cutoff, the two strains were considered to represent independent species within the genus *Rubrivirga*. Phylogenetic analyses based on 16S rRNA gene sequences consistently demonstrated strains IMCC43871^T^ and IMCC45206^T^ formed a robust clade with the three *Rubrivirga* species, supported by high bootstrap values (Fig. 1), thereby confirming their taxonomic affiliation with the genus *Rubrivirga*. The congruence among treeing methods and consistently high bootstrap values across major nodes suggest that the phylogenetic positions of the novel strains are robustly resolved.

### Phylogenomic analysis and genome characteristics

The complete genome sequence of strain IMCC43871^T^ was assembled into two contigs comprising a circular chromosome and a 28,518 bp plasmid, whereas the genome of strain IMCC45206^T^ was assembled into a single circular chromosome. The chromosomal genome sizes were 4,173,969 bp for strain IMCC43871^T^ and 4,167,368 bp for strain IMCC45206^T^, with G + C contents of 71.8% and 72.8%, respectively. CheckM analysis estimated the genome completeness and contamination at 98.4% and 1.9% for both strains, respectively, indicating a high-quality genome assembly with over 95% completeness and less than 5% contamination.

A total of 3,620 and 3,593 protein-coding sequences, 58 and 54 tRNA genes, and three rRNA genes were identified in strains IMCC43871^T^ and IMCC45206^T^, respectively. The complete 16 rRNA genes extracted from the genomes were identical (100%) to the amplified 16S rRNA gene sequences of the corresponding strains. An overview of the general genomic features of IMCC43871^T^, IMCC45206^T^, *R. marina* SAORIC-28^T^ and *R. profundi* SAORIC-476^T^ is provided in Table S1.

**Fig. 2.**
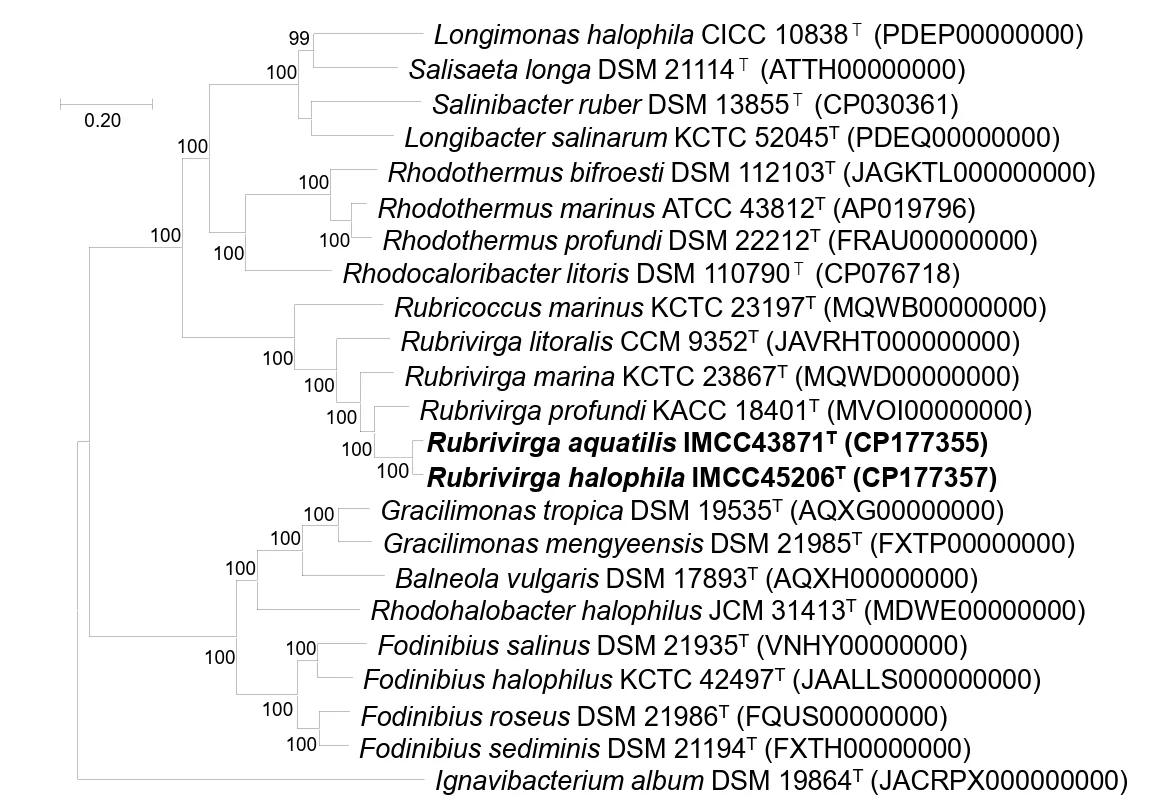
Phylogenomic tree based on concatenated multiple alignments of 81 genes showing the positions of strains IMCC43871^T^ and IMCC45206^T^. Calculations of bootstrap values are shown at nodes. Bar, 0.2 substitutions per amino acid position.

Genomic relatedness among IMCC43871^T^, IMCC45206^T^, and *Rubrivirga* species were assessed by calculating ANI and dDDH values. The ANI and dDDH values between IMCC43871^T^ and IMCC45206^T^ were 88.5% and 36.3%, respectively, below the established thresholds of 95−96% for ANI and 70% for dDDH proposed for bacterial species demarcation (Chun et al., 2018; Riesco and Trujillo, 2024), confirming that each strain represents a distinct novel species. Strain IMCC43871^T^ exhibited ANI values of 77.5% with *R. marina* SAORIC-28^T^, 77.4% with *R. profundi* SAORIC-476^T^, and 77.2% with *R. litoralis* F349^T^, while the corresponding dDDH values were 20.8%, 21.4%, and 21.3%, respectively. Similarly, IMCC45206^T^ showed ANI values of 77.7%, 77.4%, and 77.6%, and dDDH values of 21.3%, 21.4%, and 21.2% with *R. marina* SAORIC-28^T^, *R. profundi* SAORIC-476^T^, and *R. litoralis* F349^T^, respectively. All these values were well below the species delineation thresholds of ANI and dDDH, supporting the classification of strains IMCC43871^T^ and IMCC45206^T^ as two novel species within the genus. In the genome-based phylogenetic tree, strains IMCC43871^T^ and IMCC45206^T^ formed a distinct and well-supported clade within the genus *Rubrivirga*, further supporting their taxonomic assignment into the genus *Rubrivirga* (Fig. 2).

Functional annotation revealed that the genomes of IMCC43871^T^ and IMCC45206^T^ encode core metabolic pathways associated with central carbon metabolism, including the Entner–Doudoroff pathway, tricarboxylic acid cycle, pentose phosphate pathway, and phosphoribosyl pyrophosphate biosynthesis, showing the typical heterotrophic lifestyle. Both strains also harbor gene clusters related to flagellar biosynthesis; however, flagella were not observed under transmission electron microscopy. Strains IMCC43871^T^ and IMCC45206^T^ exhibited overall COG functional category distributions similar to those of other *Rubrivirga* species, but relatively higher proportions in categories such as coenzyme transport and metabolism, lipid transport and metabolism, and secondary metabolism may indicate adaptations to environmental fluctuations typical of coastal habitats (Table S2).

### Physiological and chemotaxonomic characteristics

Transmission electron microscopy revealed that cells of strains IMCC43871^T^ and IMCC45206^T^ were irregular short rods, with approximate dimensions of 0.7–0.9 µm × 1.4–2.4 µm and 0.7–0.9 µm × 1.6–2.4 µm, respectively (Fig. S1). Although genome annotation indicated the presence of genes associated with flagellar biosynthesis in both strains, flagella were not observed in the TEM images. The physiological and biochemical characteristics of strains IMCC43871^T^ and IMCC45206^T^, along with those of two closely related *Rubrivirga* species, are summarized in Table 1 and the species protologues. Strains IMCC43871^T^ and IMCC45206^T^ exhibited similar physiological traits, including optimal growth conditions, oxidase and catalase activities, and the ability to hydrolyze Tween 20 and Tween 80, but differed from each other in several enzyme activities. In addition, both strains could be distinguished from the closely related species of the genus *Rubrivirga* by differences in growth properties and certain enzymatic activities.

The fatty acid compositions of IMCC43871^T^, IMCC45206^T^, and two *Rubrivirga* species are presented in Table 2. The fatty acid profiles of the four strains were generally similar, with all strains containing C_17 :1_ ω8c and summed feature 9 (iso-C_17:1_ ω9c and/or C_16:0_ 10-methyl) as predominant components, reflecting their affiliation within the same genus. Strains IMCC43871^T^ and IMCC45206^T^ shared major fatty acids present at > 10%, including C_17 :1_ ω8c (22.3% and 20.4%, respectively) and summed feature 9 (25.2% and 25.4%), indicating close relatedness between the two novel strains. However, the two novel strains exhibited notable differences from *R. marina* and *R. profundi*, particularly in the proportions of certain fatty acids such as iso-C_17:0_ and summed feature 3 (C_16:1_ ω7c and/or C_16:1_ ω6c). These compositional variations in branched-chain fatty acids and specific unsaturated fatty acids further differentiate the two novel strains from previously described *Rubrivirga* species.

The respiratory quinone detected in both novel strains was menaquinone-7 (MK-7). The major polar lipids of strain IMCC43871^T^ included phosphatidylethanolamine (PE), phosphatidylglycerol (PG), diphosphatidylglycerol (DPG), three unidentified phospholipids, and four unidentified lipids (Fig. S2). Similarly, strain IMCC45206^T^ contained PE, PG, DPG, two unidentified phospholipids, and five unidentified lipids. Both strains shared the presence of PE, PG, and DPG, consistent with the lipid profiles reported for other *Rubrivirga* species. Despite sharing PE, PG, and DPG as major polar lipids, the novel strains differed from previously described *Rubrivirga* species in possessing distinct sets and numbers of unidentified lipids. The fatty acid composition, respiratory quinone type, and polar lipid profiles of strains IMCC43871^T^ and IMCC45206^T^ support their chemotaxonomic placement within the genus *Rubrivirga*.

### Taxonomic conclusion

Collective evidence from 16S rRNA gene- and genome-based phylogenetic analyses, along with chemotaxonomic characteristics, clearly indicated that strains IMCC43871^T^ and IMCC45206^T^ are assigned to the genus *Rubrivirga*. However, the low levels of genomic relatedness, as indicated by ANI and dDDH values well below the species delineation thresholds, together with distinct phenotypic and chemotaxonomic characteristics, clearly demonstrate that these two strains represent two novel and distinct species within the genus. Accordingly, the names *Rubrivirga aquatilis* sp. nov. and *Rubrivirga halophila* sp. nov. are proposed for strains IMCC43871^T^ and IMCC45206^T^, respectively. Beyond their taxonomic novelty, the lipid-hydrolyzing activity of the two novel species suggests ecological relevance in organic matter degradation and potential utility in marine biotechnology.

### Description of *Rubrivirga aquatilis* sp. nov.

*Rubrivirga aquatilis* (a.qua'ti.lis. L. adj. *aquatic*, pertaining to water, referring to the marine origin of the type strain).

Cells are Gram-stain-negative, obligately aerobic, non-motile rods, measuring approximately 0.7–0.9 µm in width and 1.4–2.4 µm in length. Colonies grown on marine agar are pink, circular, and convex. Growth occurs at 15–30°C (optimum, 25°C), pH 7.0–8.0 (optimum, pH 7.0), and in the presence of 1.0–4.0% (w/v) NaCl (optimum, 2.0%). Both oxidase and catalase activities are positive. In API 20NE tests, the strain is negative for nitrate reduction, esculin hydrolysis, *β*-galactosidase, indole production, glucose fermentation, arginine dihydrolase, urease, and gelatinase. In API ZYM tests, activities of alkaline phosphatase, esterase (C4), esterase lipase (C8), leucine arylamidase, valine arylamidase, trypsin, *α*-chymotrypsin, acid phosphatase, naphthol-AS-BI-phosphohydrolase, and N-acetyl-*β*-glucosaminidase are positive, whereas lipase (C14), cystine arylamidase, *α*-galactosidase, *β*-galactosidase, *α*-glucosidase, *β*-glucosidase, *α*-mannosidase, *β*-glucuronidase, and *α*-fucosidase are negative. Hydrolyzes Tween 20 and Tween 80, but does not hydrolyze casein, colloidal chitin, DNA, starch, or CM-cellulose. The predominant fatty acids are summed feature 9 (comprising iso-C_17:1_ ω9c and/or C_16:0_ 10-methyl) and C_17:1_ ω8c. The major respiratory quinone is menaquinone-7 (MK-7). The polar lipid profile includes phosphatidylethanolamine, phosphatidylglycerol, diphosphatidylglycerol, three unidentified phospholipids, and four unidentified lipids. The type strain is IMCC43871^T^ (= KCTC 102072^T^ = NBRC 116463^T^ = HNIBRBA6996^T^), isolated from coastal seawater in the Republic of Korea. The complete genome of the type strain is 4,173,969 bp in length, with a DNA G + C content of 71.8%. The GenBank accession numbers for the 16S rRNA gene sequence and genome sequence are PV186484 and CP177355, respectively.

### Description of *Rubrivirga halophila* sp. nov.

*Rubrivirga halophila* (ha.lo'phi.la. Gr. *hals*, salt; Gr. *philos*, loving; N.L. fem. adj. *halophila*, salt-loving, referring to the marine, moderately halophilic nature of the species).

Cells are Gram-stain-negative, obligately aerobic, non-motile, short rod-shaped, measuring approximately 0.7–0.9 µm in width and 1.6–2.4 µm in length. Colonies formed on marine agar are pale pink, circular, soft, and convex. Growth occurs at 20–30°C (optimum, 25°C), at pH 7.0–8.0 (optimum, pH 7.0), and in the presence of 0.5–5.0% (w/v) NaCl (optimum, 2.0%). Both oxidase and catalase activities are positive. In API 20NE tests, esculin hydrolysis, gelatinase, and *β*-galactosidase are positive, whereas nitrate reduction, indole production, glucose fermentation, arginine dihydrolase, and urease are negative. In API ZYM tests, activities of alkaline phosphatase, esterase (C4), esterase lipase (C8), leucine arylamidase, valine arylamidase, *α*-chymotrypsin, acid phosphatase, naphthol-AS-BI-phosphohydrolase, and N-acetyl-*β*-glucosaminidase are positive. No activity is detected for lipase (C14), cystine arylamidase, trypsin, *α*-galactosidase, *β*-galactosidase, *α*-glucosidase, *β*-glucosidase, *α*-mannosidase, *β*-glucuronidase, or *α*-fucosidase. Hydrolyzes Tween 20 and Tween 80, but does not hydrolyze casein, colloidal chitin, DNA, starch, or CM-cellulose. The predominant fatty acids are summed feature 9 (comprising iso-C_17:1_ ω9c and/or C_16:0_ 10-methyl) and C_17:1_ ω8c. The major respiratory quinone is menaquinone-7 (MK-7). The polar lipid profile includes phosphatidylethanolamine, phosphatidylglycerol, diphosphatidylglycerol, two unidentified phospholipids, and five unidentified lipids. The type strain is IMCC45206^T^ (= KCTC 92925^T^ = NBRC 116172^T^ = CCTCC AB 2023136^T^ = HNIBRBA14924^T^), isolated from coastal seawater in the Republic of Korea. The complete genome of the type strain is 4,167,368 bp in length, with a DNA G + C content of 72.8%. The GenBank accession numbers for the 16S rRNA gene and the genome sequence are PV076729 and CP177357, respectively.

## Figures and Tables

**Table 1.** Differential phenotypic characteristics of strains IMCC43871^T^ and IMCC45206^T^ and closely related type strains of the genus *Rubrivirga*. Strains: 1, IMCC43871^T^; 2, IMCC45206^T^; 3, *R. marina* SAORIC-28^T^; 4, *R. profundi* SAORIC-476^T^. Data were obtained from this study. All strains were cultured under identical conditions. All strains are positive for oxidase, catalase, alkaline phosphatase, esterase (C4), esterase lipase (C8), leucine arylamidase, valine arylamidase, acid phosphatase, naphthol-AS-BI-phosphohydrolase, and N-acetyl-*β*-glucosaminidase. +, positive; ‒, negative

Characteristics	1	2	3	4
Cell size (µm)	0.7–0.9 × 1.4–2.4	0.7–0.9 × 1.6–2.4	0.2–1.2 × 0.7–5.3	0.6–0.8 × 1.8–3.0
Colony color	pale pink	pink	pale red	pale red
Growth at/in				
Optimum temperature (℃)	25	25	25–30	25
pH range	7.0–8.0	7.0–8.0	6.0–9.0	6.0–8.5
Optimum pH	7	7	6.0–8.0	7.5
NaCl range (%, w/v)	1–4	0.5–5	1–5	1–5
API 20NE				
Esculin hydrolysis	‒	+	+	+
Gelatinase	‒	+	+	+
*β*-Galactosidase (PNPG)	‒	+	+	+
API ZYM				
Crystine arylamidase	‒	‒	‒	+
Trypsin	+	‒	‒	‒
*β*-Glucuronidase	‒	‒	‒	+
*α*-Chymotrypsin	+	+	‒	‒

**Table 2.** Cellular fatty acid compositions of strains IMCC43871^T^ and IMCC45206^T^ and the closely related type strains of the genus *Rubrivirga*. Strains: 1, IMCC43871^T^; 2, IMCC45206^T^; 3, *R. marina* SAORIC-28^T^; 4, *R. profundi* SAORIC-476^T^. Data were obtained from this study. All strains were cultured under identical conditions. Fatty acids comprising < 1.0% of the total fatty acid content in all species were omitted. ‒, Not detected; Tr, traces (< 1.0%). Major fatty acids (> 10%) are shown in bold.

Fatty acid (%)	1	2	3	4
**Saturated**				
C_16:0_	5.5	4.5	3.8	5.6
C_17:0_	4.2	7.9	3.9	4.7
**Branched**				
iso-C_14:0_	–	–	1.5	–
iso-C_15:0_	5.5	3.2	6.3	**12.0**
iso-C_16:0_	1.8	1.7	1.5	1.9
iso-C_17:0_	6.0	9.6	**16.1**	**15.1**
anteiso-C_15:0_	Tr	Tr	1.9	–
anteiso-C_17:0_	1.1	Tr	2.1	–
**Unsaturated**				
C_15:1_ ω6c	Tr	Tr	1.3	3.8
C_16:1_ ω9c	5.0	2.5	4.3	4.4
C_15:1_ ω8c	2.2	1.1	–	–
C_17:1_ ω6c	3.2	3.8	6.7	7.2
C_17:1_ ω8c	**22.3**	**20.4**	**12.6**	**12.7**
C_18:1_ ω9c	3.9	3.2	4.7	–
iso-C_15:1_ F	1.1	Tr	–	–
Summed feature^*^				
3	7.6	4.6	**13.6**	8.7
9	**25.2**	**25.4**	**20.0**	**23.8**

* Summed feature 3 consisted of C_16:1_ ω7c and/or C_16:1_ ω6c and summed feature 9 consisted of iso-C_17:1_ ω9c and/or C_16:0_ 10-methyl.

## Supplementary Information

Table S1. Comparative genomic features of strains IMCC43871^T^, IMCC45206^T^, *Rubrivirga marina* SAORIC-28^T^, and *Rubrivirga profundi* SAORIC-476^T^ Strains: 1, IMCC43871^T^; 2, IMCC45206^T^; 3, *R. marina* SAORIC-28^T^; 4, *R. profundi* SAORIC-476^T^

Table S2. Distribution of genes in general COG categories in the genomes of strains IMCC43871^T^, IMCC45206^T^, *R. marina* SAORIC-28^T^, and *R. profundi* SAORIC-476^T^ Strains: 1, IMCC43871^T^; 2, IMCC45206^T^; 3, *R. marina* SAORIC-28^T^; 4, *R. profundi* SAORIC-476^T^

Fig. S1. Transmission electron micrographs of cells of strains IMCC43871^T^ (A), IMCC45206^T^ (B) after negative staining with 0.5% (w/v) uranyl acetate. Cells were grown on marine agar at 25°C for 7 days. Bar, 0.2 μm.

Fig. S2. Two-dimensional thin layer chromatograph of polar lipids of strains IMCC43871^T^ (A) and IMCC45206^T^ (B). Total lipids were detected by spraying with 5% molybdatophosphoric acid solution. PE, phosphatidylethanolamine; PG, phosphatidylglycerol; DPG, diphosphatidylglycerol, PL1-3, unidentified phospholipid; L1-5, unidentified lipid. 1, first dimension of TLC; 2, second dimension of TLC.
