# Supplementary material for: Rubrivirga aquatilis sp. nov. and Rubrivirga halophila sp. nov., isolated from Korean coastal surface seawater
Source: J Microbiol. 2025 Aug 13;63(8):e2504017. doi: 10.71150/jm.2504017 (PMC13577104; doi:10.71150/jm.2504017)

Fig. S1. Transmission electron micrographs of cells of strains IMCC43871^T (A), IMCC45206^T (B) after negative staining with 0.5% (w/v) uranyl acetate. Cells were grown on marine agar at 25°C for 7 days. Bar, 0.2 μm.

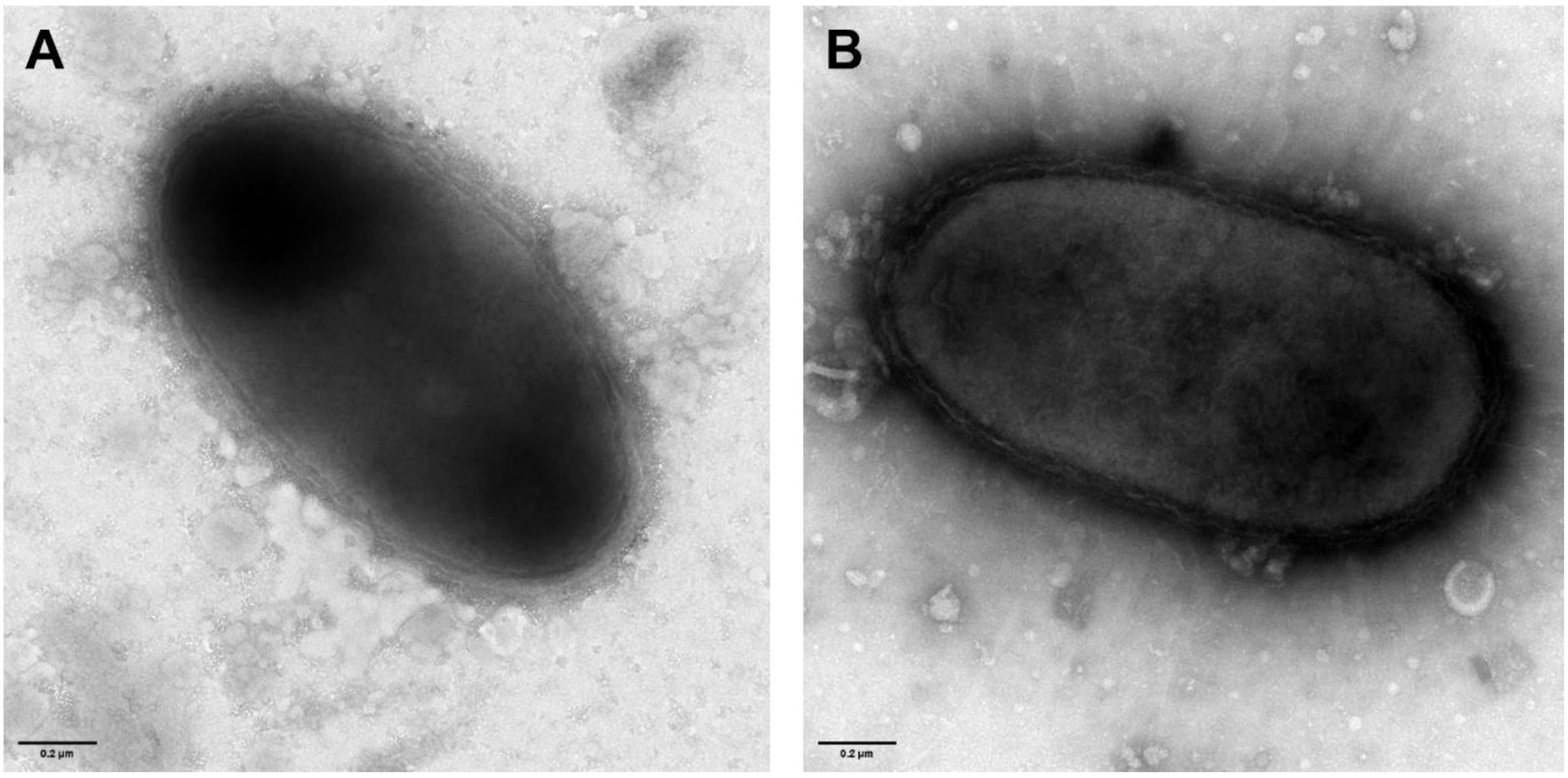

Supplement: Supplementary file 1 [file jm-2504017-Supplementary-Fig-S1.pdf]
