# Supplementary material for: Rubrivirga aquatilis sp. nov. and Rubrivirga halophila sp. nov., isolated from Korean coastal surface seawater
Source: J Microbiol. 2025 Aug 13;63(8):e2504017. doi: 10.71150/jm.2504017 (PMC13577104; doi:10.71150/jm.2504017)

Fig. S2. Two-dimensional thin layer chromatograph of polar lipids of strains IMCC43871^{T} (A) and IMCC45206^{T} (B). Total lipids were detected by spraying with 5% molybdatophosphoric acid solution. PE, phosphatidylethanolamine; PG, phosphatidylglycerol; DPG, diphosphatidylglycerol, PL1-3, unidentified phospholipid; L1-5, unidentified lipid. 1, first dimension of TLC; 2, second dimension of TLC.

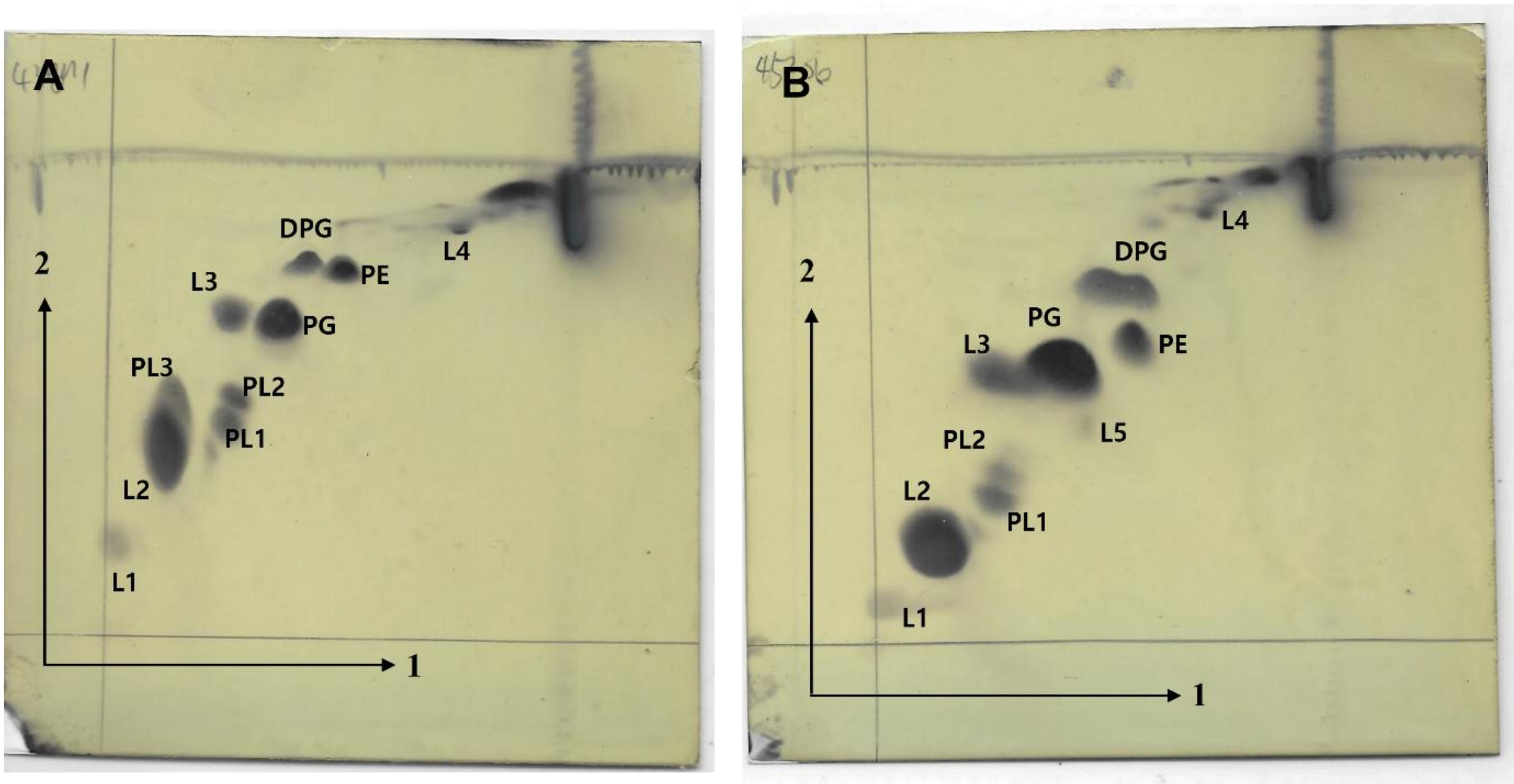

Supplement: Supplementary file 2 [file jm-2504017-Supplementary-Fig-S2.pdf]
