# Supplementary material for: Rubrivirga aquatilis sp. nov. and Rubrivirga halophila sp. nov., isolated from Korean coastal surface seawater
Source: J Microbiol. 2025 Aug 13;63(8):e2504017. doi: 10.71150/jm.2504017 (PMC13577104; doi:10.71150/jm.2504017)
Supplement: Supplementary file 3 [file jm-2504017-Supplementary-Table-S1.pdf]

Table S1. Comparative genomic features of strains IMCC43871^T, IMCC45206^T, *Rubrivirga marina* SAORIC-28^T, and *Rubrivirga profundi* SAORIC-476^T

Strains: 1, IMCC43871^T; 2, IMCC45206^T; 3, *R. marina* SAORIC-28^T; 4, *R. profundi* SAORIC-476^T

	1	2	3	4
**Genomic stats**				
GenBank Accession number	CP177355	CP177357	MQWD00000000*	MVOI00000000*
Size (bp)	4,173,969	4,167,368	4,981,075	4,601,673
Number of contigs	2	1	11	41
G + C content (%)	71.8	72.8	72.5	71.5
Number of tRNA	58	54	56	49
Number of rRNA	3	3	3	3
Protein coding sequence	3,620	3,593	4,254	3,892
**Genome validation (based on CheckM)**				
Completeness	98.4	98.4	97.3	98.4
Contamination	1.9	1.9	2.5	2.5

* Genomes of *R. marina* SAORIC-28^T and *R. profundi* SAORIC-476^T were downloaded from NCBI GenBank database and annotated in this study for comparative genomic analysis.
