# Supplementary material for: Rubrivirga aquatilis sp. nov. and Rubrivirga halophila sp. nov., isolated from Korean coastal surface seawater
Source: J Microbiol. 2025 Aug 13;63(8):e2504017. doi: 10.71150/jm.2504017 (PMC13577104; doi:10.71150/jm.2504017)
Supplement: Supplementary file 4 [file jm-2504017-Supplementary-Table-S2.pdf]

Table S2. Distribution of genes in general COG categories in the genomes of strains IMCC43871^T, IMCC45206^T, *R. marina* SAORIC-28^T, and *R. profundi* SAORIC-476^T

Strains: 1, IMCC43871^T; 2, IMCC45206^T; 3, *R. marina* SAORIC-28^T; 4, *R. profundi* SAORIC-476^T

Code	Description	1	2	3	4
**J**	Translation	7.2	6.9	6.1	6.9
**A**	RNA processing and modification	-	-	-	-
**K**	Transcription	4.5	4.2	4.7	4.6
**L**	Replication, recombination and repair	4.3	4.1	4.6	4.1
**B**	Chromatin structure and dynamics	0.1	0.1	0.1	0.1
**D**	Cell cycle control, cell division, chromosome partitioning	1.4	2.4	1.7	2.5
**V**	Defense mechanisms	2.6	2.6	2.5	2.3
**T**	Signal transduction mechanisms	8.1	7.9	7.2	7.6
**M**	Cell wall/membrane/envelope biogenesis	9.0	8.7	8.5	8.7
**N**	Cell motility	1.8	1.9	1.6	1.0
**Z**	Cytoskeleton	-	0.1	-	-
**W**	Extracellular structures	-	-	-	-
**U**	Intracellular trafficking, secretion, and vesicular transport	2.8	2.3	2.6	2.7
**O**	Posttranslational modification, protein turnover, chaperones	5.1	5.6	5.2	4.9
**X**	Mobilome: prophages, transposons	0.3	0.2	0.7	0.6
**C**	Energy production and conversion	4.9	5.1	4.7	4.7
**G**	Carbohydrate transport and metabolism	4.4	4.6	7.5	6.3
**E**	Amino acid transport and metabolism	7.0	6.3	6.3	6.1
**F**	Nucleotide transport and metabolism	2.4	2.5	2.2	2.5
**H**	Coenzyme transport and metabolism	5.3	5.3	4.4	4.7
**I**	Lipid transport and metabolism	4.6	4.7	4.3	4.5
**P**	Inorganic ion transport and metabolism	4.9	5.0	6.1	5.3
**Q**	Secondary metabolites biosynthesis, transport and catabolism	2.1	2.1	1.9	1.9
**R**	General function prediction only	10.0	10.3	9.6	9.6
**S**	Function unknown	7.0	7.2	7.4	8.3
